# Supplementary figures and images for: A new species of the genus Sarsamphiascus Huys, 2009 (Copepoda: Harpacticoida: Miraciidae) from a sublittoral zone of Hawaii
Source: PeerJ. 2020 Feb 10;8:e8506. doi: 10.7717/peerj.8506 (PMC7017794; doi:10.7717/peerj.8506)

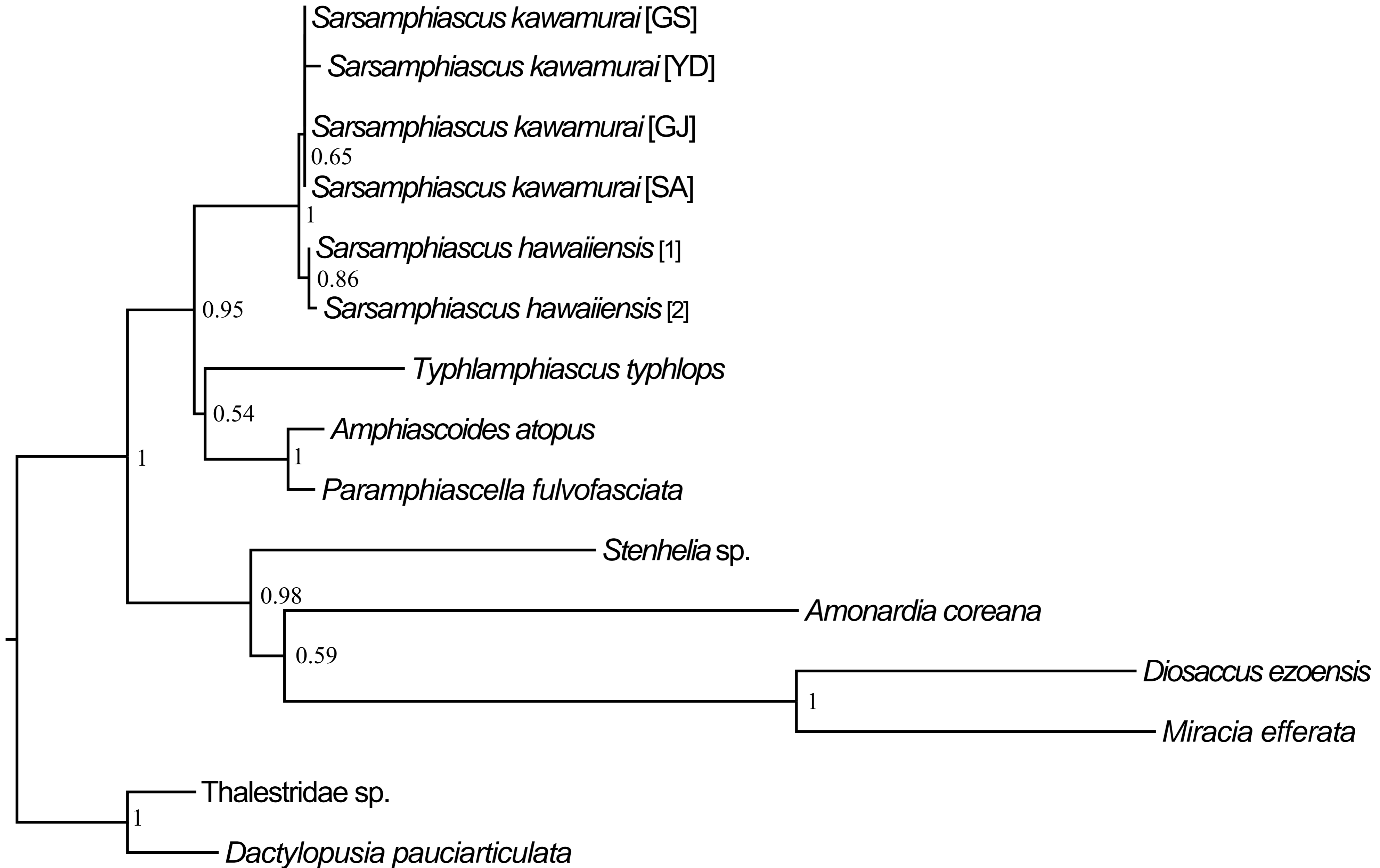

0.01

Supplement: Figure S1 [file peerj-08-8506-s004.pdf]
